# Scientific Items

**Published:** 1883-07-20

**Authors:** 


### SCIENTIFIC ITEMS.

**How to Act in a Tornado.**—Sergeant John P. Finley, signal service officer at Kansas City, Missouri, has published, in a pamphlet on tornadoes, some useful directions concerning the course to be taken to escape the dangers of those terrible forces. The inhabitant of a tornado-frequented district must be watchful in the season of visitations, for he can never know when the destruction will come upon him. On the first sign of the approaching vortex he must run, always to the north, unless by going in that direction he will have to cross the entire path of the storm. If he is nearer to the southern edge than to the centre of the probable path, he may go south, bearing slightly east; but in no event should he ever run directly to the east or northeast. It is impossible to save any building that may lie in the path of the tornado, or any property that cannot be got out of its way. No material, no method of construction can be competent to resist the raging destruction. Nothing rising above the ground can escape it. The most practicable measure of precaution is to construct a "dug-out" at some suitable point, within easy distance from the house, to serve as a place of refuge or shelter. The retreat should be entirely underground, with a roof at least three feet thick, not rising above the surface of the earth, and entered from the northern or eastern side. A "cellar-cave" may be coustructed from the cellar, if the house has one, to serve as a substitute for the "dug-out." It should be excavated from the west wall of the cellar, toward the west, and should be made as complete and secure as the "dug-out." If, however, the storm can not be escaped, if no refuge is at hand, or there is not time to get to it, the safest thing to do is to place one's self against the west wall of the cellar, face forward, or against the south wall, as near the southwest corner as possible. The northeast quarter is, in any case, a fatal position, and should always be avoided. If one is actually overtaken by the tornado, his only resource is to cast himself face downward upon the ground, with his head to the east and his arms thrown over his head to protect it. If a stump or large stone, or anything heavy that the wind will not blow over is near, he may get a trifle of protection by throwing himself to the eastward of it. If in a house with no cellar, he should get into the west room on the ground-floor if possible, and away from all stoves and heavy furniture. The people of towns might find it to their advantage to provide for having a watch, to be on duty on all days when the air bears the premonitory symptoms of a violent windstorm, to give a signal to the whole population on the appearance of the first real threatening signs. The signs of the formation and approach of a tornado-cloud are distinct and sufficiently suggestive to afford opportunity for timely and concerted action, Sergeant Finley is continuing his investigations of the phenomena of tornadoes, and he has prepared three full schedules of minute inquiries, calling for the facts attendant upon the appearance of the storms, which he sends to persons who were within the path of one, who were on the outer edge of the path, and who were from ten to one hundred miles from it.—*Popular Science Monthly.*

**Photography and Pock-marks.**—Dr. Hermann Vogel, in his work entitled "The Chemistry of Light and Photography," published in the International Scientific Series, states that there are faces with little yellowish specks that do not strike the eye, but which come out very dark in photography. A few years ago a lady was photographed in Berlin whose face had never presented specks in photography. To the surprise of the photographer, on taking her portrait, specks appeared that were invisible in the original. A day later the lady sickened of small-pox, and the specks, at first invisible to the eye, became then quite apparent. Photography in this case had detected before the human eye the pock-marks, very feebly tinged yellow.—*Lancet.*

**Cheap Water Filter.**—Very many families desire some inexpensive device for filtering rain and other waters to be used for cooking and table use. A cheap and very efficient filter may be made by using a spirit or wine cask, placing it on end, with the head removed, and having a faucet at the bottom to draw off the clear water. To fit it for a filter, take the removed top head of the cask, and with a small bit bore holes all over it, then place four clean bricks or blocks of wood on the bottom and on these rest the perforated top. Now fill upon it about four inches of charcoal chopped into small bits the size of peas, and over this place a layer of clean sand, six inches deep. Impure water poured into the cask on top of the sand will become clear and sparkling after a little while, or as soon as all fine particles are worked out of the charcoal and sand. This filter, will not need renewing oftener than once in two or three months.—*Ibid.*

**Sun-Spot Laws.**—From a careful re-examination of 120 years' records of sun-spots, M. Wolf, of Zurich, comes to these three conclusions:

(1.) There is a period of 10 years. (2.) There is a second period of 11 years, 4 months. (3). There is not a period of 12 years, imputable to the action of Jupiter. Notwithstanding the great difference of the two periods, the interval from a minimum to the following maximum is the same for both, namely, 4 1-2 years. After 170 years, the maxima and minima are reproduced in the same order and with the same numerical value.—*Pop. Science News.*

**A Mastodon Graveyard.**—The city of Dallas, Texas, is said to be built over a graveyard of mastodons, and for five or six years past excavations for buildings have seldom failed to bring up their bones. A large number of these mastodon remains were unearthed recently, and some of the bones were of enormous size.—*Ibid.*

